# Preliminary results of a French multi-centre audit on transmission-based precautions

**DOI:** 10.1186/2047-2994-4-S1-P91

**Published:** 2015-06-16

**Authors:** M Giard, M-A Ertzscheid, N Garreau, E Caillat-Vallet, N Vernier, D Landriu, C Laland, D Zaro-Goni, A Savey, M Aupée

**Affiliations:** 1 CClin Sud-Est, Hospices Civils de Lyon, Lyon, France; 2 Université Lyon 1, Villeurbanne, France; 3 CClin Ouest, Rennes, France; 4 CClin Sud-Est, Lyon, France; 5 CClin Est, Nancy, France; 6 CClin Paris-Nord, Paris; 7 CClin Sud-Ouest, Bordeaux, France

## Introduction

In addition to standard precautions, transmission-based precautions (TBP) aim to prevent cross transmission of micro-organisms from patient to patient or to healthcare workers (HCW). In France, guidelines were updated in 2009 for contact precautions and in 2013 for droplet and airborne precautions.

## Objectives

The objective was to assess TBP implementation in healthcare settings (HCS).

## Methods

The study consisted in a mixed audit of procedures, resources and knowledge. It was conducted between January 1^st^ and December 31^st^ 2014. Inclusion criteria were voluntary public and private hospitals in France. Self-assessment questionnaires were administered at two levels: institutional and HCW. At ward level, an external auditor observed how TBP were applied for each patient requiring them (organisation, supplies and environment). Data were computed online by each HCS. Results were given as a percentage of objectives attained at institutional and ward levels, and as percentages of correct answers attained at HCW level. Pooled scores were calculated by criteria.

## Results

A total of 547 hospitals participated, including 6,148 patients requiring TBP, 129,514 HCW and 5,114 physicians. At institutional level, the alert system score was 81.5%. In particular, 99.1% of HCS had a procedure to follow concerning internal information in case of multidrug resistant bacteria (MDRB), 60.3% had an information system in case of re-admission of a MDRB patient. Moreover, 84.6% of HCS had a procedure to check whether TBP were implemented in case of MDRB, 91.6% of whom traced it. At ward level, 95.0% of the implemented TBP belonged to the appropriate category. The overall score was 84.2%, going from 68.6% for prescription traceability to 92.2% for room organisation. Further analyses are ongoing and these results will be available in June 2015 (HCW level, stratifications by speciality).

## Conclusion

Such a large participation will serve as a national reference leading to TBP promotion actions for security improvement at local and national levels, as announced in the French national programme on healthcare-associated infections prevention.

## Disclosure of interest

None declared.

